# Rosai-Dorfman Disease in a Middle-Aged Man With Recurrent Pleural Effusions and Lymphadenopathy

**DOI:** 10.7759/cureus.64825

**Published:** 2024-07-18

**Authors:** Zaheer Aslam, Omer Chowdhary, Anjana Razik, Mostafa Negmeldin

**Affiliations:** 1 Respiratory Medicine, Bedford Hospital NHS Trust, Bedford, GBR; 2 Respiratory Medicine, Luton and Dunstable University Hospital, Luton, GBR; 3 General Internal Medicine, Bedford Hospital NHS Trust, Bedford, GBR

**Keywords:** rosai-dorfman disease, h-caldesmon, cd68, bcl-2, s-100, pegylated interferon 90, hilar lymphadenopathy, histiocytes, extra nodal

## Abstract

Rosai-Dorfman disease (RDD) is a rare benign condition that presents most commonly with lymphadenopathy and skin lesions and is characterized by infiltration of histiocytes into the skin and soft tissues. We present a case of RDD in an Afro-Caribbean male in his 50s who presented to our chest clinic with shortness of breath, cough, and weight loss of 15 kg over one year. CT scan showed evidence of right pleural effusion, mediastinal and hilar lymphadenopathy, and bony lesions in the spine. Cytology from multiple pleural effusions and endobronchial ultrasound-guided fine needle aspiration from lymph nodes did not show any malignancy. Left axillary excisional biopsy showed a pattern consistent with RDD. The patient was started on interferon therapy by the hematologist and pleurodesis after repeated pleural taps failed to relieve recurrent right pleural effusions. This case emphasizes the importance of tissue diagnosis to avoid misdiagnosis and unnecessary treatment.

## Introduction

Rosai-Dorfman disease (RDD) is a rare disorder characterized by the abnormal accumulation of histiocytes and massive lymphadenopathy, with a prevalence of approximately one per 200,000 people in the United States [1]. It is also known as Destombes-Rosai-Dorfman disease. It is named after Pierre-Paul Louis Lucien Destombes who first described this disease in four patients in 1965 and pathologists Juan Rosai and Ronald Dorfman who described it first as "sinus histiocytosis with massive lymphadenopathy." The abnormal histiocytes may accumulate in the lymph nodes causing lymphadenopathy or may be found in other areas of the body, including skin, subcutaneous tissues, and visceral organs. The clinical presentation associated with RDD varies and depends on the extent of the disorder and the organs or organ systems affected. Patients may present with painless lymphadenopathy or may have non-specific symptoms like fever, malaise, pallor, chronic rhinitis, headache, night sweats, and joint pain. In certain cases, the presentation may mimic malignancy, posing diagnostic challenges due to the presence of symptoms and imaging findings consistent with malignancies. Thus, it is imperative to confirm the diagnosis through histopathological examination of tissue samples. The exact cause of RDD is unknown.

## Case presentation

A man in his 50s presented to the accident and emergency unit with a four-day history of bilateral painful red eyes, accompanied by a right-sided temporal headache and blurred vision in both eyes. He had a past medical history of diabetes mellitus controlled by oral hypoglycemics. He was a nonsmoker and denied any alcohol consumption. He also denied any exposure to toxic fumes or chemicals. Examination showed bilaterally inflamed, red conjunctivae. The rest of the eye examination and systemic examinations were otherwise normal. Lab results showed a high CRP of 40 and an erythrocyte sedimentation rate of 120. The rest of the blood tests were unremarkable. He was discharged on a high dose of steroids after a provisional diagnosis of temporal arteritis was made and subsequently referred to the rheumatologist. However, the possibility of temporal arteritis was ruled out by the rheumatologist after repeated normal ultrasounds of the temporal artery. A chest X-ray done as part of the workup was normal. As the redness in the eyes persisted, he underwent a right conjunctival biopsy that showed an inflammatory process unusual for that site.

Four months later, he presented to the general practitioner (GP) with concerns about a lump in the left axilla persisting for one month. An ultrasound-guided core biopsy of the lump was performed. The biopsy report revealed generous cores of tissue incorporating some entrapped fat, a diffuse proliferation of bland-appearing spindle cells with relatively abundant cytoplasm, and cells with a somewhat histiocytoid appearance and foci of foamy cells in some areas. A relatively diffuse lymphoid population was observed, with some areas aggregating into nodules. Plasma cells were conspicuous in certain areas. There was no evidence of cytological atypia, mitotic activity, or necrosis. The abnormal histiocytic cells on immunohistochemistry demonstrated positivity for S-100 protein, CD4, and CD68 (PGM1), with strong nuclear cyclin D1 positivity and OCT2 positivity. They were negative for CD1a, melan-A, soft tan (sic), cytokeratin AE1/AE3, CD21, CD23, CD34, and desmin. There was widespread positivity for S-100, both in the more spindled and histiocytoid areas. Bcl-2 exhibited strong diffuse positivity present in both the spindle cell areas and the lymphoid areas. CD68 showed focal weak positivity. Smooth muscle actin (SMA) and H-caldesmon highlighted the vessels within the specimen, with some weak focal positivity in certain spindled areas. CD34 highlighted the vessels exclusively. CD138 highlighted the presence of abundant plasma cells, which showed polytypic kappa and lambda light chain expression consistent with a reactive plasmacytic infiltrate. While the observed appearances could potentially represent a reactive process, their unusual nature prompted a referral for a specialist soft tissue pathology opinion, specifically to exclude the possibility of a low-grade sarcomatous process.

**Figure 1:**
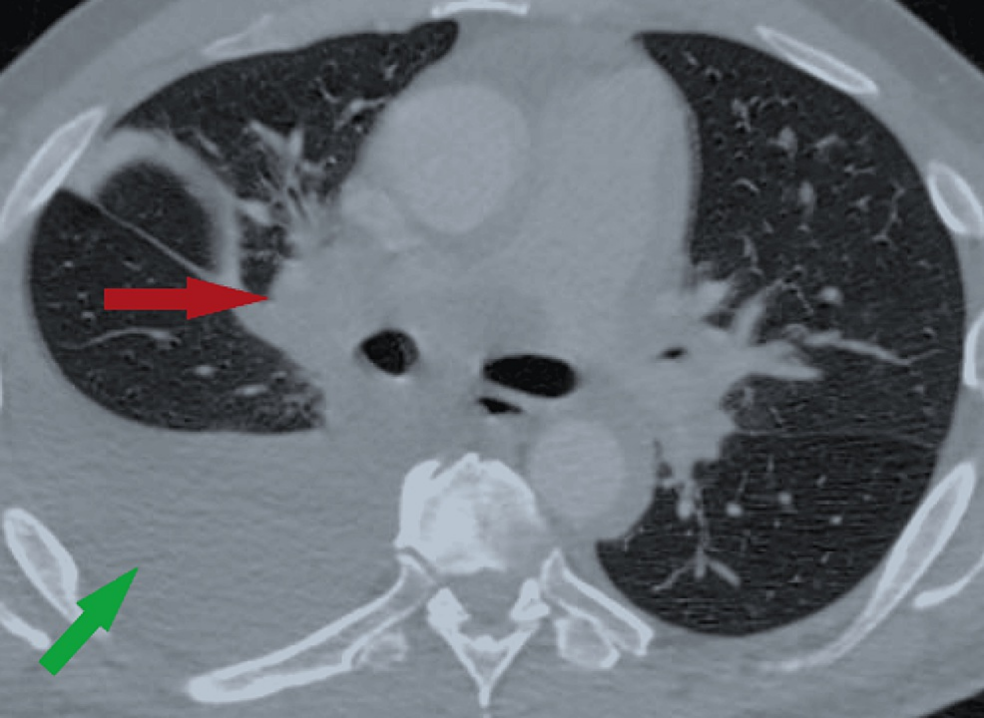
Contrast-enhanced CT scan of the thorax. The green arrow points to a very large right pleural effusion occupying most of the right pleural space. There is no significant left pleural effusion. The red arrow points to a large right perihilar mass with some irregularity.

**Figure 2:**
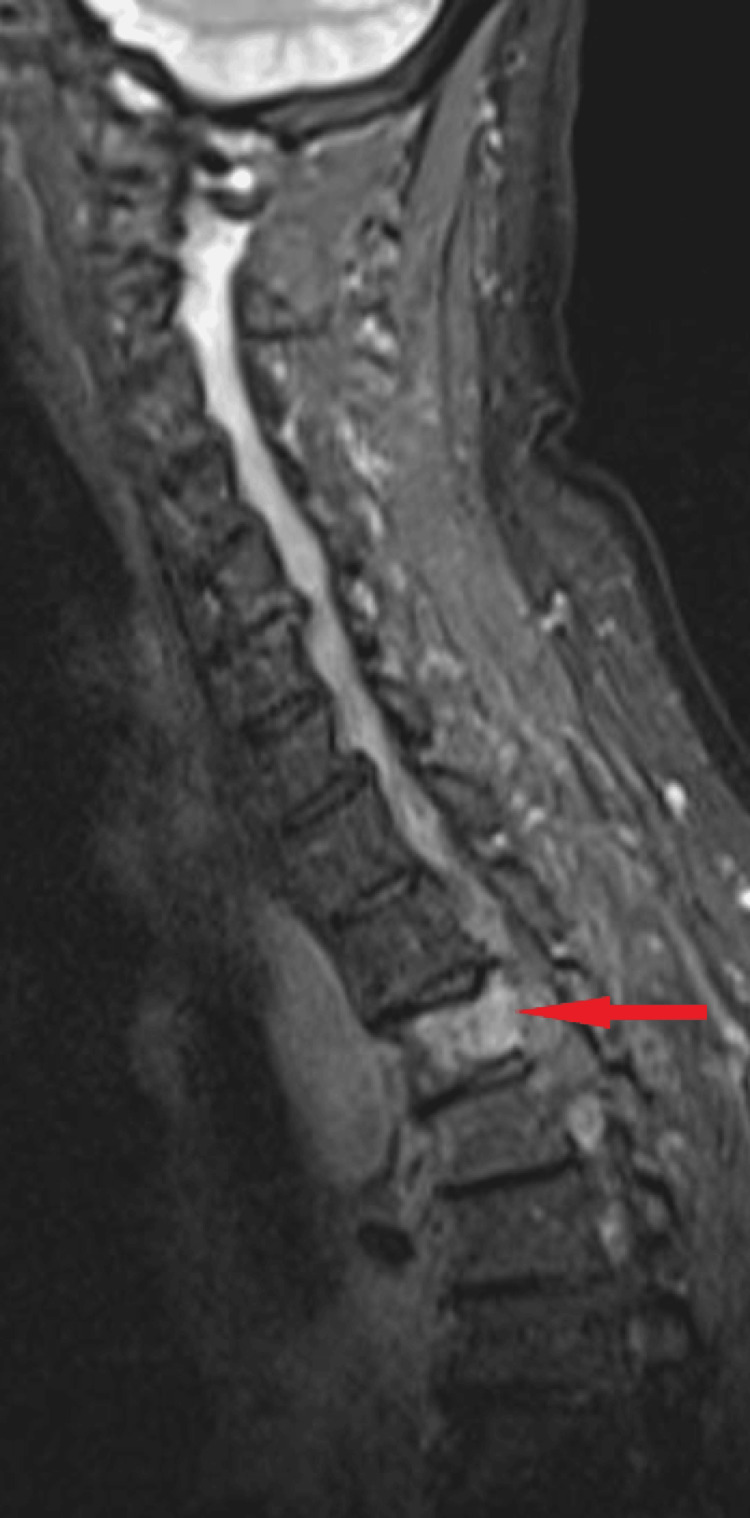
Magnetic resonance imaging of the spine. The red arrow points to an area of hyperintensity in the T3 vertebra. The report suggested that this was likely to be malignant infiltration with cord compression. There was a diffusely abnormal marrow signal throughout the cervical and thoracic spine on T1-weighted imaging.

**Figure 3:**
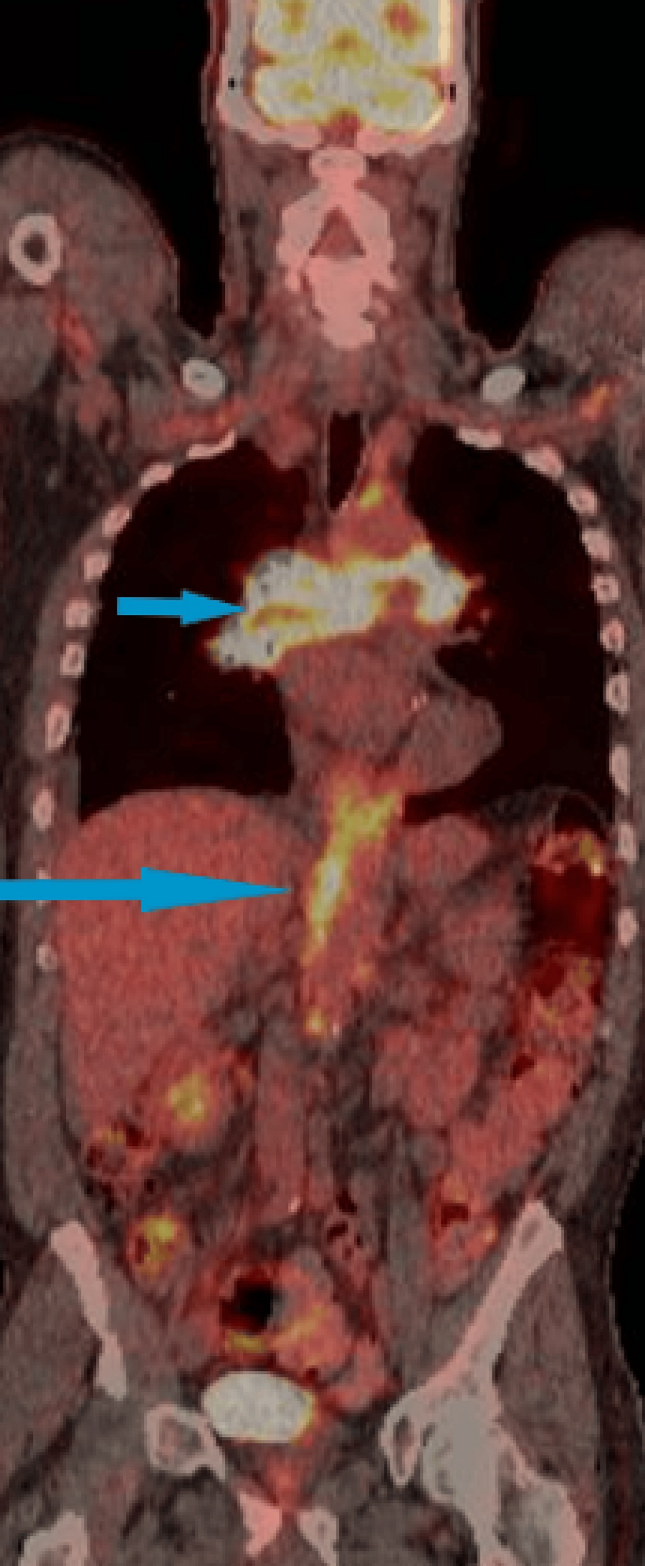
Fludeoxyglucose-18 (FDG) positron emission tomography (PET) scan. Blue arrows point to increased FDG uptake in both lung hila more on the right side and in soft tissue extending around the descending aorta at the level of the diaphragm. There was increased FDG uptake in the lung hila (SUV max = 13.5) and in soft tissue extending around the descending aorta with an SUV max of 12.0 at the level of left hilum and 12.5 at the level of the diaphragm. SUV max: maximum standardized uptake value.

**Figure 4:**
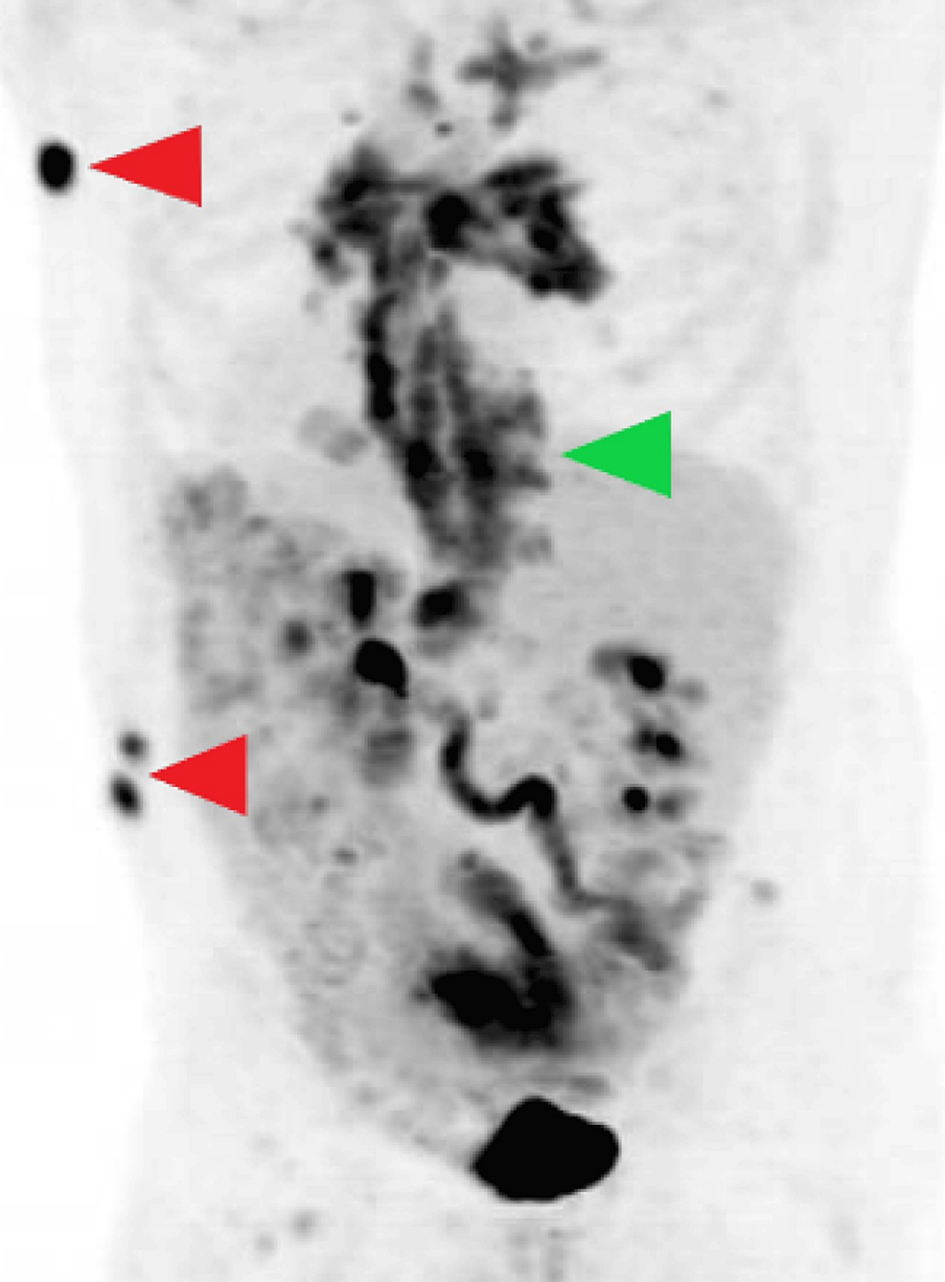
Fludeoxyglucose-18 (FDG) positron emission tomography (PET) scan. The green arrow points to FDG uptake associated with lytic destruction of T3, which has an SUV max of 8.9. Uptake extends into the spinal canal between T2 and T4. The red arrows point to multiple FDG-avid subcutaneous nodules. A 3 cm subcutaneous nodule inferiorly in the left axilla has an SUV max of 16.5. Two nodules in the left flank have an SUV max of 12.4. SUV max: maximum standardized uptake value.

Five months following the biopsy, he presented to his GP with complaints of a cough of two months duration and shortness of breath. The cough had been intermittent, accompanied by a weight loss of approximately 15 kg and a diminished appetite. He was a non-smoker and worked in a warehouse with no significant occupational exposures. He denied experiencing neurological, abdominal, or joint symptoms. Chest examination revealed bilateral wheeze. The chest X-ray ordered by the GP revealed a mildly elevated right hemidiaphragm with a small pleural effusion. The lungs appeared clear otherwise. Given the X-ray findings and the patient's presenting symptoms, a CT scan was conducted and he was referred to the chest clinic under the suspected cancer pathway. Baseline investigations, including liver and kidney functions, electrolytes, autoimmune screen, and echocardiography were unremarkable. The CT scan of the thorax, abdomen, and pelvis with contrast revealed a large mass in the right hilar region, along with extensive conglomerate lymphadenopathy within the mediastinum and bilaterally within the hila (Figure 1). Furthermore, it indicated multilevel degenerative discogenic changes of the vertebrae, along with mixed cystic and sclerotic alterations within the vertebral body of T3, suggestive of a hemangioma or metastasis. Additionally, a focal area of sclerosis with a central low-density region was observed within the left mid-lower iliac bone, just below the left sacroiliac joint. Further investigation with MRI was recommended to provide clarity. MRI of the whole spine showed a T3 lesion likely to be malignant with early cord compression (Figure 2). PET-CT showed appearances suggestive of widespread metastatic lesions. However, no primary tumor was identified (Figures 3, 4).

The patient was reviewed in the chest clinic. A pleural aspiration and endobronchial ultrasound (EBUS) were arranged. Pleural aspiration was negative for malignancy even when repeated but revealed an exudative pattern with mesothelial cells, numerous foamy macrophages, small lymphocytes, and a few plasma cells. EBUS was performed twice and reported multiple bilateral mediastinal and hilar lymphadenopathy. The lymph node in station 7 was described as large, round, and vascular with an indistinct margin, heterogeneous echogenicity, absent central structure, and absent coagulation necrosis. Histopathology showed no evidence of granulomatous inflammation or malignancy.

Other differential diagnoses included lymphoma, tuberculosis, and sarcoidosis. Ziehl-Neelsen stain was negative for tuberculosis.

The findings were discussed in the multi-disciplinary team (MDT) meeting. Lung cancer was highly suspected and ruled out by pleural cytology and histopathology. The expert tissue pathology received for the axillary mass was reviewed in the MDT meeting. Expert opinion remarked that the features are consistent with extranodal (possibly cutaneous) Rosai-Dorfman-Destombes disease. OCT2 and cyclin D1 positivity supported the diagnosis, and a recent study has suggested OCT2 as a novel marker for the Rosai-Dorfman macrophage/monocyte phenotype [2]. It should be noted that RDD can be associated with immune disorders, IgG4-related disorders, neoplasia, or even inherited conditions. Histologically, there was no significant increase in IgG4-positive plasma cells in the sample.

Based on the histopathology reports, he was diagnosed with extranodal RDD. The patient received a course of high-dose steroids and radiotherapy to the involved vertebrae for early cord compression. The patient underwent talc pleurodesis for recurrent right pleural effusion. The hematologist commenced the patient on pegylated interferon at a dose of 90 micrograms weekly, which he tolerated well. The patient is under the care of the hematology team currently and the disease is well-controlled with weekly interferon treatment. There is no new site of disease involvement. The patient complains of tiredness, which is a common adverse effect of interferon therapy. He can perform normal activities of daily life; however, his exercise tolerance has reduced.

## Discussion

RDD, also known as sinus histiocytosis with massive lymphadenopathy, is a rare condition. It is more common among individuals of Afro-Caribbean descent and typically manifests in the first and second decades of life [3]. RDD is now part of the "R group" of histiocytosis, which includes familial RDD, sporadic RDD, and other miscellaneous non-cutaneous, non-Langerhans cell histiocytosis. It may be sporadic or familial. Sporadic RDD is the most prevalent type, which includes the classic nodal form, extranodal RDD, neoplasia-associated RDD, and immune disease-associated RDD. The extranodal form may involve bones or a single organ or can be disseminated. RDD may be associated with neoplasia like leukemia, lymphoma, malignant histiocytosis, Langerhans cell histiocytosis, and Erdheim-Chester disease. It may also be associated with autoimmune diseases like systemic lupus erythematosus, idiopathic juvenile arthritis, autoimmune hemolytic anemia, and HIV. A specific form of isolated RDD, known as cutaneous RDD, has been identified, affecting only the skin. Cutaneous RDD is separately classified under the "C group" of histiocytoses [4].

The most common presentation includes bilateral massive painless cervical lymphadenopathy, which may be the only symptom. In some cases, there may be additional nonspecific symptoms like fever, malaise, pallor, and weight loss. Approximately 40% of patients experience extranodal involvement, affecting areas such as the skin, nasal cavity, bones, orbits, and central nervous system. Cutaneous lesions are typically characterized as papulonodular, plaques, papules, and xanthoma-like. Pulmonary involvement is poorly described. Mediastinal lymphadenopathy is the most common intrathoracic manifestation. Other manifestations include pleural effusion, interstitial lung disease, and pulmonary mass [5]. There are also reports of laryngotracheal involvement causing obstructive symptoms like stridor and hoarseness.

The role of macrophage colony-stimulating factor has been considered as a possible trigger, and viruses like human herpes simplex may contribute to the pathogenesis. Additionally, RDD may be associated with HIV, lymphoma, amyloidosis, and other lymphoproliferative disorders [6]. Prognosis varies, with reported deaths alongside favorable outcomes due to the benign nature of the disease. Histopathology plays a central role in diagnosis. Emperipolesis, a phenomenon in which histiocytes engulf lymphocytes and possibly other types of cells, is commonly observed. Immunohistochemical staining is positive for S100, CD68, and CD163, but negative for CD1a stain. Sporadic cases of RDD are usually self-limiting, with spontaneous remission reported in up to 50% of cases. However, up to 10% of patients may succumb to direct complications, infections, or amyloidosis [7]. Observation is recommended for patients with uncomplicated adenopathy and asymptomatic cutaneous disease. Surgical excision may be necessary for unifocal extranodal disease or symptomatic airway, cranial, spinal, or sinus involvement. In cases of multifocal unresectable extranodal disease, systemic therapy may be required, although there is currently no standardized regimen. Available systemic therapies include corticosteroids, sirolimus, radiotherapy, chemotherapy, and immunomodulatory therapy.

## Conclusions

Classic RDD is part of the "R group" of histiocytosis and cutaneous RDD is part of the "C group." RDD is not a malignancy and does not have the concept of "staging" commonly used in malignancies. The severity of the condition is determined by the extent of the spread. A thorough medical history, physical examination, and laboratory and radiological investigations are required to establish the diagnosis and extent of the disease. Obtaining tissue biopsies or samples from multiple sites significantly increases diagnostic yield and therefore accuracy. A comprehensive approach is crucial in patient management and underscores the importance of a multi-disciplinary strategy that involves relevant specialties. Notably, in cases involving lymphadenopathy and pleural effusion, it is imperative to consider histiocytic disorders within the differential diagnoses. Spontaneous remission of RDD occurs in many cases and can be managed with observation alone, whereas some patients may require steroids, surgery, chemotherapy, or immunomodulatory agents.
